# Short- and Long-Term Evaluation of a Fixed Dose of *Beauveria bassiana* Strain MS-8 Diluted in Various Doses of Kaolin as a Powder Formulation Applied to Rice Grains to Control Almond Moth, *Ephestia cautella* Walker (Lepidoptera: Pyralidae)

**DOI:** 10.3390/microorganisms10101971

**Published:** 2022-10-05

**Authors:** Mohamed Baha Saeed, Mark D. Laing, Ray M. Miller

**Affiliations:** 1 Discipline of Plant Pathology, School of Agricultural, Earth and Environmental Science, University of KwaZulu-Natal, Durban 4001, South Africa; 2 Faculty of Agriculture, Department of Crop Protection, University of Khartoum, Khartoum 11115, Sudan; 3 School of Biological and Conservation Science, University of KwaZulu-Natal, Durban 4001, South Africa

**Keywords:** *Beauveria bassiana*, post-harvest, grain pests, biological control, *Ephestia cautella*

## Abstract

Short-term and long-term evaluation studies were conducted against *Ephestia cautella* on rice grains, using *Beauveria bassiana* Strain MS-8 formulated in various doses of kaolin as an active carrier. In the short-term study (45 days), a fixed dose of 0.03 g conidia kg^−1^ of grain of Strain MS-8 was formulated in kaolin at doses of 0.3, 0.5, 1, and 2 g kg^−1^ of grain. These formulations were evaluated for their effects on larval mortality and the number of adults emerged. The highest level of larval mortality (90.0%) and the lowest numbers of adults emerged (1.6 insect/100 g of rice grain) were caused by Strain MS-8 in a kaolin dose of 2 g kg^−1^. However, Strain MS-8 in a kaolin dose of 1 g kg^−1^ performed well for the same parameters. In the long-term evaluation study (180 days), the same dose of Strain MS-8 was formulated in kaolin at doses of 0.5, 1, 2, and 3 g kg^−1^ of grain. These formulations were then evaluated against the levels of webbed grain, grain damage, and weight loss. The lowest levels of webbed grain (2.0%), grain damage (3.0%), and weight loss (1.8%) were caused by Strain MS-8 in kaolin at a dose of 3 g kg^−1^, although Strain MS-8 in kaolin doses of 1 g and 2 g kg^−1^ also performed well for the same parameters. The highest levels of webbed grain (15.0%), grain damage (30.0%), and weight loss (9.0%) were observed in the untreated control treatment (UCT).

## 1. Introduction

Insect pests cause global losses to stored grains, quantitatively and qualitatively, particularly in tropical and subtropical countries [1,2]. It is estimated that insects destroy between 10.0% and 30.0% of all food produced in Africa each year [3,4]. The assessments of rice yield losses due to insects in Africa range between 10.0% and 15.0% [5]. This loss differs regionally, by country and by rice variety, and in some years may exceed 90.0% [6].

The almond moth, *Ephestia cautella* Walker (Lepidoptera: Pyralidae), is a pest of a wide range of commodities, including cereals and cereal products, cocoa, and oilseeds. It is found throughout the tropics and subtropics [7]. The larvae are the damaging form of this insect, as they feed on the whole grains and the seed germ [8]. The webbing and the frass produced by the larvae of this pest affect the esthetics and handling of infested produce. The insect also causes physical damage to grain, enhancing infection by *Aspergillus* species that release mycotoxins [9].

The fumigation and application of neurotoxic contact insecticides have been widely used to protect stored grains. In the case of fumigants, the most commonly used product, phosphine, has lost its efficacy in some areas. Several species of stored-grain insects have developed a significant level of resistance to this compound, making its application ineffective in many parts of the world [10,11,12,13]. Several stored-grain insects have developed resistances to commonly used pyrethroid and organophosphate insecticides [14,15,16,17]. The development of resistance to chemical insecticides and concerns over the adverse effects of chemicals on the environment and human health have provided the stimulus for developing microbial control agents for use in the integrated control of pests of stored grains.

Entomopathogenic fungi (EPF) represent one of the most promising alternatives to chemical control against stored-grain insects [18,19]. *Beauveria bassiana* (Balsamo) Vuillemin (Ascomycota: Hypocreales) is the most widely tested EPF for the control of stored-grain insects [20,21,22,23]. The dried conidia of EPFs may be formulated with various natural dusts, such as diatomaceous earth, charcoal, oven ash, and chalk powder [24,25,26,27]. However, little is known about the use of other natural dusts, such as kaolin, as carriers of the dried conidia of EPFs to control *E. cautella* in rice. Therefore, the objective of this study is to evaluate the effects of a fixed dose of *B. bassiana* Strain MS-8 with various doses of kaolin as powder formulations applied to rice grains to control almond moth *E. cautella* in a one-generation, 45 d trial, and in a six-month trial.

## 2. Materials and Methods

### 2.1. Insect rearing

The initial stock culture of *E. cautella* used in this study was obtained from the Department of Plant Pathology, through the School of Agricultural, Earth and Environmental Science (SAEES) at the University of KwaZulu-Natal. Samples of 1 kg of yellow maize grain (Smith Animal Feed, Unit 8 Davlen Park, 11 Halsted Rd, Mkondeni, Pietermaritzburg, South Africa, 3201. The seed morphology was intermediate between flint and dent, and the source was Brazil, but the variety was unknown) was introduced into 6 L plastic boxes (Basix Plastics, 400 Victoria Road, Pietermaritzburg, South Africa). Ten couples of insects (one day old) were released into the boxes. Each box was covered with insect nets (Filmflex Plastics Natal cc, Unit 10, Shepstone Park, Corner of Shepstone and Blasé Roads, New Germany, South Africa). This was to allow for adequate aeration and to prevent moth escape. The culture was maintained under the experimental conditions (28 ± 2 °C and 65 ± 5% RH) until a new generation of adult insects emerged. Emerged insects were used for the experiments.

### 2.2. Fungus and Dry Conidia Preparations

The fungus *B. bassiana* Strain MS-8 used in this study was isolated from soil samples from Ukulinga Research Farm, Pietermaritzburg, South Africa using *Galleria mellonella* L. (Lepidoptera: Pyralidae) as live insect bait (Zimmermann, [28]).

The fungus was identified morphologically according to Humber [29], using a light microscope (Zeiss Axiophot) and with PCR by Inqaba Biotech, South Africa (https://inqababiotec.co.za, accessed on 18 July 2022). The primers used were ITS1 (5′-TCCGTAGGTGAACCTGCGG-3′) and ITS 2 (5′-GCTGCGTTCTTCATCGATGC-3). The accession number with Inqaba Biotech was NR 111594.1.

A procedure documented by Gouli et al. [30], with modifications, was used to prepare the dry conidia. The initial production of the fungus was conducted on potato dextrose agar (PDA) for 14 days at 28 °C. Mature conidia were collected from the surface of the medium. A conidial suspension was adjusted to 1 × 10^8^ conidia mL^−1^ by using a Neubauer Improved Hemocytometer (Hirschmann®, Pietermaritzburg, South Africa). Rice grains were washed well (1.5 kg) and soaked overnight. The rice grains were dispensed into three sterile autoclavable bags (Whitehead Scientific (Pty) Ltd, Unit 9, Van Biljon Industrial Park, Winelands Close, Strickland, 7530) (305 × 660 mm). Each bag was filled with 500 g of grain. The bags and rice gains were then sterilized at 121 °C for 15 min. followed by a 24 h cooling period at 22–25 °C. Fifty milliliters of fungal suspension were used to inoculate the sterilized rice grains in the plastic bags using a medical syringe. After inoculation, the contents of the bags were mixed to ensure an even distribution of the fungal suspension among the grains and kept for 15 days at 25 °C. Each bag was closed using a special stopper, having a diameter of 10 cm and length of 6 cm, which formed the aeration mouth. The mouth of the bag was covered by a special stopper consisting of three layers of paper, tissue, and aluminum foil, which faced the exterior of the bag. The first two layers protected the nutrient substrate from contamination, and the third layer prevented the premature drying of the cultivated material for the first four days. Four days after inoculation, the upper parts of the aluminum foil were removed to facilitate air circulation. The bag stoppers were opened after ten days to expedite fungal sporulation. After 15 days, the fungal biomass was spread evenly into paper towels in a layer of 1 cm deep and air-dried for 4–7 days under laminar flow. The dried conidia were harvested by sieving the infested grains through a 100 mm diameter sieve (150 µm pore size) and kept at 4 °C for further use.

### 2.3. Grain Preparations

White rice grains (Milled white rice, Osman’s Taj Mahal, 180 Sirdar Road, Clairwood, Durban, South Africa) were obtained from a local supermarket. To remove any hidden infestation by insects, the grains were kept in a freezer at −20 °C for one week.

### 2.4. Effect of B. bassiana Powder Formulations on Larval Mortality and Adults Emerged in the Short-Term Trial

*B. bassiana* Strain MS-8 at a dose of 0.03 g conidia kg^−1^ of grain was formulated in kaolin (Kaolin White from Serina Trading (www.kaolin.co.za accessed 2 September 2022)), which has a chemical composition of SiO_2_ (45%) + Al_2_O_3_ (36%) with trace amounts of Fe_2_O_3_, TiO_2_, CaO, MgO, Na_2_O, and K_2_O, at doses of 0.3, 0.5, 1, and 2 g kg^−1^ of grain. All treatments of *B. bassiana* were mixed with kaolin, which was used as the neutral carrier. Kaolin is an excellent carrier of Bb because of its low moisture content, its neutral action on Bb, and a slight enhancement of the activity of Bb relative to other carriers such as corn flour. This may be due to its slightly abrasive action on the cuticle of insects. A zero level Bb (i.e., 100% kaolin) control was not run because prior research had shown that kaolin at 2 g kg^−1^ caused mortality levels of around 20% in the target pests (Saeed, [31]), a result similar to that found by others (Salerno et al. [32]). The use of the same dose of kaolin for all treatments, combined with Abbott’s Correction, ensured that the slight insecticidal effect of kaolin was corrected to zero for all treatments. The specific mixtures of kaolin and conidia of *Beauveria bassiana* Strain MS-8 were combined in a Petri dish, which was then sealed with parafilm, and mixed well by shaking by hand.

These powder formulations were tested for their effects on *E. cautella* larvae. For each treatment, three glass jars of 250 mL, each containing 100 g of rice grains, were used as replicates of each treatment. The control treatment was left untreated. Subsequently, a male and female newly emerged adult were introduced into each glass jar, which were then covered with insect nets. Adult insects were removed after the oviposition period of one day. After eggs hatched, larvae were examined for mortality every 3 days for a period of 21 days. The number of adults that emerged after 45 days of storage was recorded. To confirm the fungus as the cause of larval death, the dead larvae were surface sterilized with 70% sodium hypochlorite, followed by rinsing three times in distilled water, then placed in Petri dishes lined with filter paper. The Petri dishes were kept in an incubator at 28 °C. Only the larval counts showing fungal growth were considered for larval mortality due to fungus.

### 2.5. Persistence of B. bassiana Powder Formulations against E. cautella in the Long-Term Trial

*B. bassiana* Strain MS-8 at a dose of 0.03 g conidia kg^−1^ was formulated in kaolin at doses of 0.5, 1, 2, and 3 g kg^−1^ of grain. These powder formulations were evaluated for their activity against *E. cautella* for 180 days in stored rice grains. For each formulation, three glass jars of 3 L capacity, each containing 1 kg of rice grains, were used as replicates. Formulated conidia and rice grains were mixed manually for three min to achieve an even distribution in the powder. Two pairs of newly emerged adults (one day old) were released in each jar, which were then covered with insect nets for air circulation. Adults were left to oviposition and died within seven to nine days. Pairs of newly emerged adults were added every 45 days (one generation) for all the treatments, including the UCT, until the end of the experiment to ensure that every treatment had a new infestation, particularly those that had strong effects on the larvae of *E. cautella*. This mimicked the arrival of new moths to infest rice grains.

### 2.6. Data Collection

Every 45 days for 180 days (six months), subsamples of 100 g of rice grains were taken from each treatment to measure the evaluation parameters. The webbed grains were collected and weighed, and then, the webbed grains were expressed as a proportion of the total weight of grains. The rice grains were then sieved, and the clean grains were weighed to calculate the weight loss from the original weight (Tefera [33]). Ten grams of rice grains from each sub-sample was taken, and the number of grains damaged by insects feeding was expressed as a percentage of the total number of grains. The grain samples and insects (larvae and adults) were returned to the respective treatments after evaluations.

(1) Weight loss (%)=(original weight−weight after 45 days)/(original weight)×100

### 2.7. Statistical Analysis

The experiments were arranged in a randomized complete blocks design. Mortalities were corrected relative to the control using Abbott’s formula [34]. Data were subjected to analysis of variance (ANOVA) using GenStat for Windows, 17th edition [35]. Means were separated using Fisher’s Least Significant Differences at the 5% level of significance. A Shapiro–Wilk test (SAS) was conducted on the data to test for normality before subjecting the data to ANOVA or Linear regression.

## 3. Results

### 3.1. Effect of B. bassiana Powder Formulations on Larval Mortality

**Figure 1:**
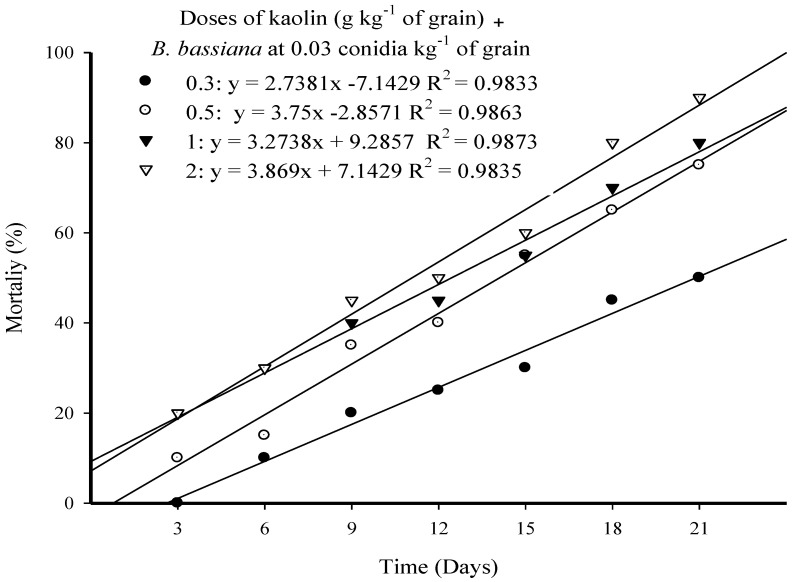
Corrected mortality (Abbott’s correction) of *Ephestia cautella* larvae following exposure to *Beauveria bassiana* Strain MS-8 at a fixed dose of 0.03 conidia g kg^−1^ formulated in kaolin at doses of 0.3, 0.5, 1, and 2 g kg^−1^ of grain.

Highly significant differences were observed for the main effects of doses (*F = 333.31; *p* < 178 0.001; *df* = 3*) and time (*F = 637.24; *p* < 0.001; *df* = 6*), and the interaction effects of doses and time (*F = 4.91; *p* < 0.001; *df* = 18*) on larval mortality. Linear regressions were calculated between time and doses on the mortality of *E. cautella* larvae with an R^2^ > 0.98 for the four doses (Figure 1).

The application of a fixed dose of 0.03 g conidia kg^−1^ of *B. bassiana* Strain MS-8 formulated in kaolin at 0.3, 0.5, 1, and 2 g kg^−1^ of grain caused mortality levels of 50.0, 75.0, 80.0, and 90.0% on *E. cautella* larvae, respectively, after 21 days of exposure. Mortality was less than 5.0% in the control treatment.

### 3.2. Effect of B. bassiana Powder Formulations on Number of Adults Emerged

**Figure 2:**
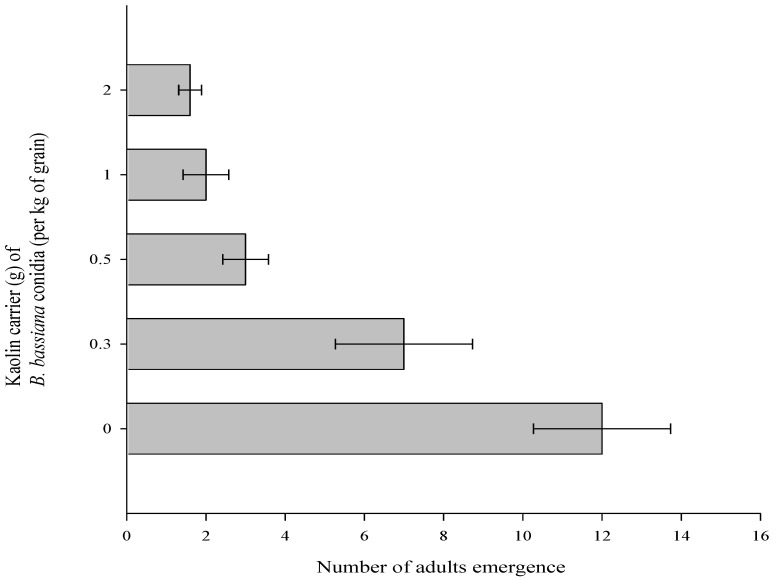
Mean number of *Ephestia cautella* adults that emerged from rice grains after 45 days of infestation following treatment with *Beauveria bassiana* Strain MS-8 at 0.03 g conidia kg^−1^ formulated in various doses of kaolin.

Highly significant differences were observed between the tested doses (*F* = 40.45; *p* < 0.001; *df* = 4). The treatment of rice grains with *B. bassiana* Strain MS-8 at all doses of kaolin significantly reduced the number of adults that emerged relative to the UCT (Figure 2). Strain MS-8 at kaolin doses of 2 g and 1 g kg^−1^ caused the lowest number of adults to emerge (1.6 and 2 adults/100 g of rice grain) compared to the number of adults emerged of 12 adults/100 g of rice grain on the UCT (Figure 2). It was observed that a small number of *E. cautella* larvae avoided contact with the fungal conidia because they created a web cocoon extremely early, into which they migrated (Supplementary Figure S1). This behavior limited their periods of feeding, and subsequently, the adults that emerged were small compared to the UCT adults (Supplementary Figure S2). These small adults laid relatively few eggs, which slowed the population growth. This behavior suggests that infected larvae communicate with uninfected larvae, triggering early cocoon formation.

### 3.3. Webbed Grain (%) in Long-Term Trial

**Figure 3:**
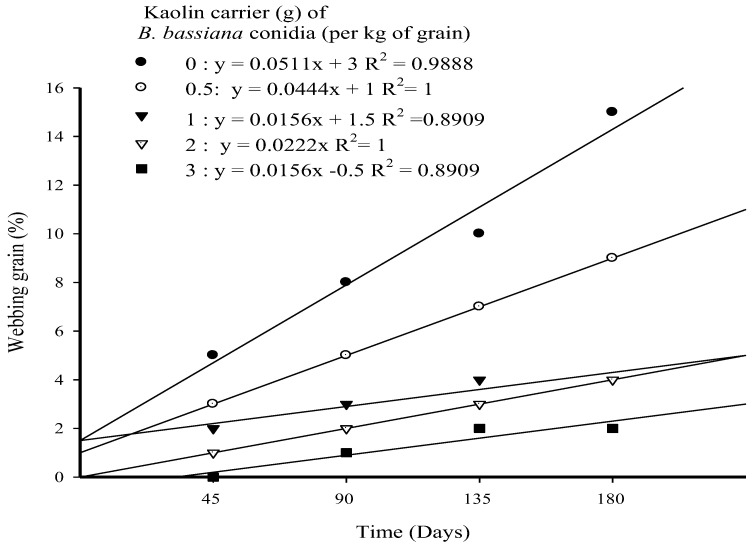
Webbed grains over 180 days of *Ephestia cautella* larvae infestation of rice following treatments with *Beauveria bassiana* Strain MS-8 at 0.03 g conidia kg^−1^ formulated in various doses of kaolin.

There were highly significant differences between the doses (*F* = 53.51; *p* < 0.001; *df* = 4) and time (*F* = 23.77; *p* < 0.001; *df* = 3) and their effects on the level of webbed grain after 180 days of storage. The interaction between doses and time (*F* = 2.64; *p* < 0.011; *df* = 12) was also significant. A linear relationship was observed between dose and time for the level of webbed grain, with an R^2^ > 0.89 (Figure 3). The treatment of rice grains with Strain MS-8 at all doses of kaolin caused significantly lower levels of webbed grain after 180 days of storage. After the first 45 days of storage (one generation of *E. cautella*), no webbed grain was observed with Strain MS-8 at a kaolin dose of 3 g kg^−1^. However, webbed grain of 5.0% was observed with the UCT. These results were the inverse of mortality (Figure 3). After 180 days of storage (four generations of *E. cautella*), Strain MS-8 at a kaolin dose of 3 g kg^−1^ caused the lowest level of the webbed grain of 2.0%, compared to a level of 15.0% for the UCT. No significant differences in the levels of webbed grain were observed for kaolin doses of 1 g and 2 g kg^−1^ by the end of the experiment (Figure 3).

### 3.4. Grain Damage (%) in Long-Term Trial

**Figure 4:**
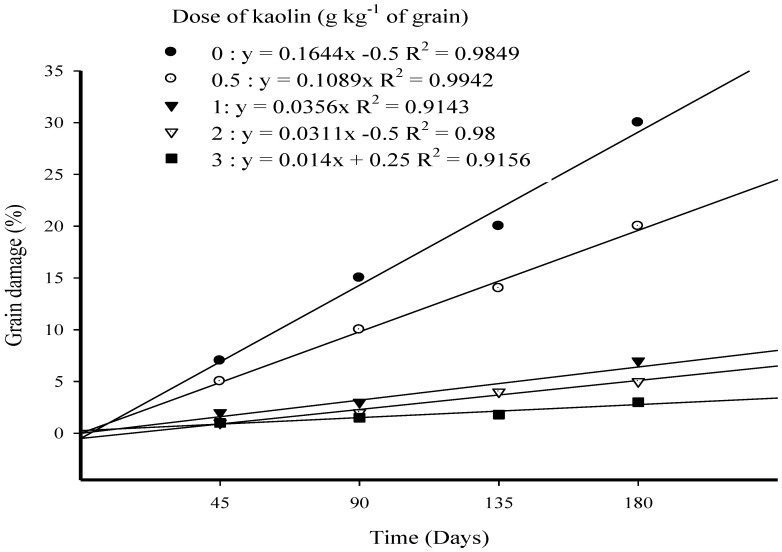
Grain damage over 180 days after *Ephestia cautella* larvae infestation on rice grains, following exposure to *Beauveria bassiana* Strain MS-8 at 0.03 g conidia kg^−1^ of grain formulated in various doses of kaolin.

Highly significant differences were observed between the main effects of doses (*F* = 162.92; *p* < 0.001; *df* = 4) and time (*F* = 70.8; *p* < 0.001; *df* = 3) and the interaction effects of doses and time (*F* = 11.58; *p* < 0.001; *df* = 12). The grain damage levels were reduced by all *B. bassiana* Strain MS-8 treatments. The application of *B. bassiana* Strain MS-8 at all doses of kaolin to rice grain caused significant reductions in the levels of grain damaged by *E. cautella* larvae (Figure 4). After 180 days of storage, the lowest level of grain damage (3.0%) was caused by Strain MS-8 at a kaolin dose of 3 g^−1^, compared to a grain damage level of 30.0% in the UCT. In contrast, highly significant differences were observed with Strain MS-8 at kaolin doses of 0.5 and 1 g kg^−1^ which reduced grain damage levels to 20.0% and 7.0%, respectively. These results indicate that kaolin improved the distribution of the fungal conidia, and therefore, more larvae were infected and killed. No significant differences were observed between the doses of kaolin 1, 2, and 3 g kg^−1^ after the first 90 days of storage (Figure 4).

### 3.5. Grain Weight Loss (%) in Long-Term Trial

**Figure 5:**
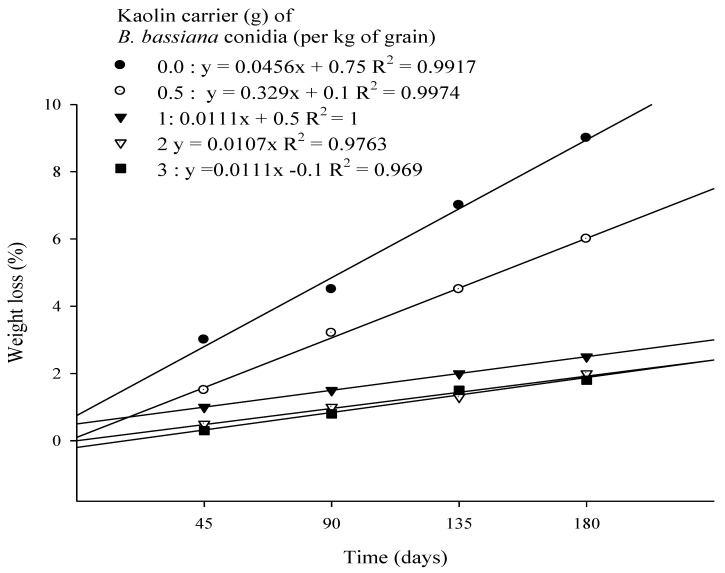
Grain weight loss over 180 days of *Ephestia cautella* larvae infestation of rice grains following treatments with *Beauveria bassiana* Strain MS-8 at 0.03 g conidia kg^−1^ of grain formulated in various doses of kaolin.

Both of the main factors caused significant differences in the levels of weight loss, namely dose (*F* = 73.22; *p* < 0.001; *df* = 4) and time (*F* = 36.35; *p* < 0.001; *df* = 3). The interaction effect between dose and time (*F* = 3.83; *p* < 0.001; *df* = 12) was also significant. Over the experimental period, a similar efficacy was observed for Strain MS-8 at kaolin doses of 2 g and 3 g on the levels of weight loss. However, at 180 days, treatment with Strain MS-8 at a kaolin dose of 3 g kg^−1^ caused the lowest weight loss of 1.8%, relative to weight loss of 9.0% in the UCT. More than double the weight loss was observed over the experimental period after treatment with Strain MS-8 at kaolin doses of 0.5 g and 1 g, except during the first 45 days, during which there was no significant difference between treatments (Figure 5). The result of the correlation analysis between mortality and secondary parameters is presented in Table 1.

## 4. Discussion

Formulations of a fixed dose of *B. bassiana* Strain MS-8 in various doses of kaolin generated a range of mortality levels in *E. cautella*. For example, the treatment of rice grains with Strain MS-8 at a kaolin dose of 0.3 g and 2 g kg^−1^ of grain caused mortality levels of 50.0 and 90.0% after 21 days of exposure, respectively. Increasing kaolin doses could have improved the distribution of the fungal conidia, and therefore, would have increased the frequency of contact between the larvae and conidia, thus increasing infestation and insect death. These results indicated that kaolin improved the efficacy of *B. bassiana* conidia, either by increasing the distribution of fungal conidia, or by abrasion of the waxy epidermis of target insects, as has been suggested by Salerno et al. [32] and Samodra and Ibrahim [36], who observed that the waxy layer of insect integuments was abraded by kaolin, which allowed for greater conidial attachment and fungal penetration through the insect exoskeleton. Samodra and Ibrahim [37] evaluated the dried conidia of eight isolates of entomopathogenic fungi on *Corcyra cephalonica* Stainton (Lepidoptera: Pyralidae) in rice grains. Two isolates of *B. bassiana* (BbGc and BbPs) and one isolate of *Metarhizium anisopliae* (MaPs) caused high mortality levels in *C. cephalonica* larvae. Isolates of BbGc and BbPs formulated in kaolin and of BbPs formulated in tapioca flour caused 100% larval mortality by the 15th day of exposure. More than 90% mortality was recorded when rice grains were stored for 4 months.

In the present study, twelve adults emerged in the UCT. However, the application of Strain MS-8 at all doses of kaolin significantly reduced the number of adults that emerged from rice grains after 45 days of storage. Kaur et al. [22] observed that the exposure of *C. cephalonica* larvae to *B. bassiana*-treated sorghum grains resulted in a significant reduction in adult emergence. Similarly, Cherry et al. [20] found that *B. bassiana* 0362 at 1 × 10^7^ and 1 × 10^8^ conidia g^−1^ of grain led to significant adult mortality and reduced F1 emergence relative to an untreated population of *Callosobruchus maculatus* (F.) (Coleoptera: Bruchidae). The present study indicated that the application of a fixed dose of *B. bassiana* Strain MS-8 formulated in various doses of kaolin not only caused mortality in *E. cautella* but also produced small adults. These behaviors suggest that infected larvae communicate with uninfected larvae, triggering early cocoon formation that ultimately leads to the suppression of the pest population. Kaur et al. [22] observed morphological deformities in the adults of *C*. *cephalonica* following exposure to *B. bassiana* treatments. This may be a survival strategy by this pest to avoid hostile conditions, including the natural incidence of entomopathogens.

Apart from direct infestation, the feces and webbing produced by the larvae of *Ephestia kuehniella* Zeller (Lepidoptera: Pyralidae) spoil the product [38]. In this study, a significant reduction in the levels of webbed grain occurred after the application of MS-8 at all doses of kaolin after 180 days of storage. The lowest level of webbed grain (2.0%) occurred after treatment with Strain MS-8 in a kaolin dose of 3 g kg^−1^. This finding indicated that either kaolin interfered with the web formation, or it improved the fungal distribution of conidia and therefore increased the chances of larvae contacting conidia, becoming infected, and dying.

The levels of grain damage and weight loss were the result of the higher or lower levels of pest populations that remained after the treatment of rice grains with Strain MS-8 at all kaolin doses. For instance, the lowest levels of grain damage (3.0%) and weight loss (1.80%) resulted from treatment with Strain MS-8 at a kaolin dose of 3 g kg^−1^, which also caused the highest level of larval mortality (90.0%) when applied at a dose of 2 g kg^−1^ of grain. In contrast, the highest levels of grain damage and weight loss were both observed in the UCT. These results agree with the findings of [37], who observed a direct relationship between levels of weight loss and the pest population after four months of *S. oryzae* infestation on rice grains, following treatment with *B. bassiana* isolates (BbGc and BbPs) formulated in kaolin. Padin et al. [39] documented that damage to wheat grains by *S. oryzae* was reduced by treatments with *B. bassiana*.

The correlation analysis clearly showed that the secondary trials measured were highly inversely correlated with the primary parameter, namely the mortality of insects. The secondary parameters were closely correlated with each other (99.3–99.9%).

In conclusion, *B. bassiana* Strain MS-8 formulated in kaolin was effective in the control of *E. cautella* in rice grains after 180 days of storage. The application of this strain could be used as a model to control other rice grain insects such as the rice moth, *C. cephalonica*. This study was conducted under controlled conditions, which differ from on-farm conditions. Therefore, further studies should be conducted to test the performance of this strain under on-farm conditions to deal with the practical challenges of providing protection to stored grain in the field.

## Figures and Tables

**Table 1:** Correlation coefficients between the measured secondary parameters and mortality of insects.

	Adult Emerged (%)	Grain Damage (%)	Webbed Grain (%)	Weight Loss (%)	Mortality (%)
Adult emerged (%)	-				
Grain damage (%)	0.993 ***	-			
Webbed grain (%)	0.994 ***	0.989 **	-		
Weight loss (%)	0.999 ***	0.996 ***	0.991 ***	-	
Mortality (%)	−0.996 ***	−0.981 **	−0.999 ***	−0.995 ***	-

* Significant at *p* = 0.05; ** significant at *p* = 0.01; *** significant at *p* < 0.00.

## Data Availability

Not applicable.

## Supplementary Materials

The following supporting information can be downloaded at: https://www.mdpi.com/article/10.3390/microorganisms10101971/s1, Supplementary Figure S1. Webs made by *Ephestia cautella* larvae to avoid hostile conditions, following treatment of rice grain with *Beauveria bassiana* MS-8 at a kaolin dose of 2g kg^−1^ of grain. Supplementary Figure S2. **A** = *Ephestia cautella* adults that emerged from untreated rice grain; **B** = adults that emerged from rice grains, following treatment with *Beauveria bassiana* MS-8 at a kaolin dose of 2g kg^−1^ of grain, showing the difference in size.
