# Supplementary material for: Short- and Long-Term Evaluation of a Fixed Dose of Beauveria bassiana Strain MS-8 Diluted in Various Doses of Kaolin as a Powder Formulation Applied to Rice Grains to Control Almond Moth, Ephestia cautella Walker (Lepidoptera: Pyralidae)
Source: Microorganisms. 2022 Oct 5;10(10):1971. doi: 10.3390/microorganisms10101971 (PMC9607579; doi:10.3390/microorganisms10101971)
Supplement: Supplementary file 1 [file microorganisms-10-01971-s001.zip › microorganisms-1840009-supplementary.pdf]

microorganisms

MDPI

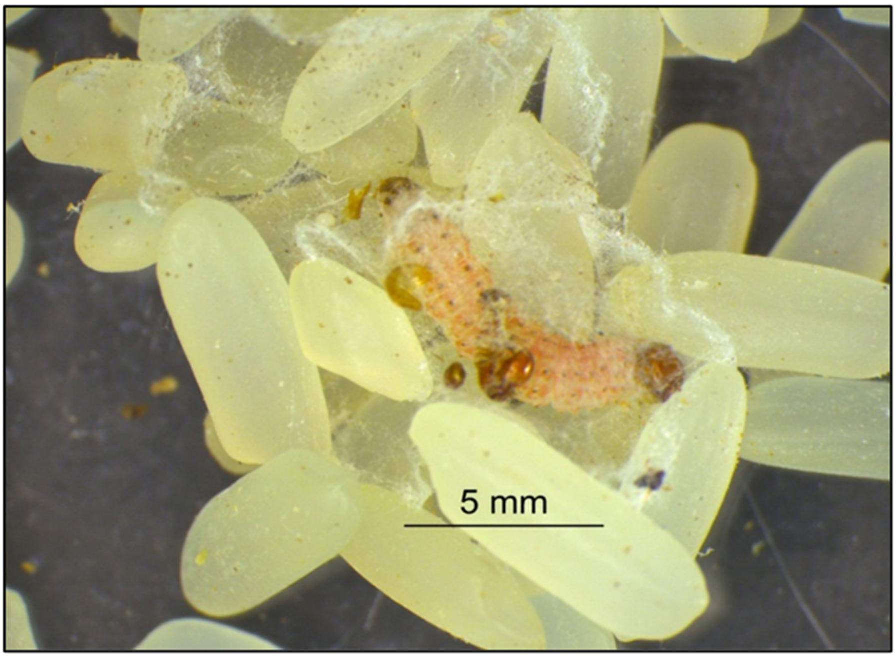

Figure S1. Webs made by *Ephestia cautella* larvae to avoid hostile conditions, following treatment of rice grain with *Beauveria bassiana* MS-8 at a kaolin dose of 2g kg^{-1} of grain

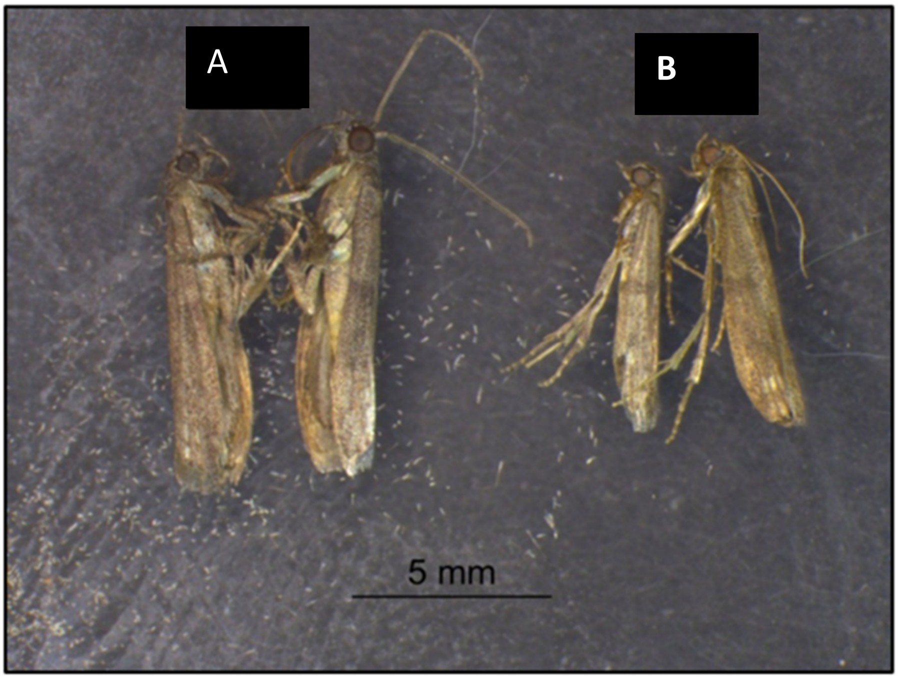

Figure S2. A= *Ephestia cautella* adults that emerged from untreated rice grain; **B** = adults that emerged from rice grains, following treatment with *Beauveria bassiana* MS-8 at a kaolin dose of 2g $kg^{-1}$of grain, showing the difference in size
